# Pd Catalysts Supported on Mixed Iron and Titanium Oxides in Phenylacetylene Hydrogenation: Effect of TiO_2_ Content in Magnetic Support Material

**DOI:** 10.3390/nano14171392

**Published:** 2024-08-26

**Authors:** Eldar T. Talgatov, Akzhol A. Naizabayev, Farida U. Bukharbayeva, Alima M. Kenzheyeva, Raiymbek Yersaiyn, Assemgul S. Auyezkhanova, Sandugash N. Akhmetova, Evgeniy V. Zhizhin, Alexandr R. Brodskiy

**Affiliations:** 1 D.V. Sokolskiy Institute of Fuel, Catalysis and Electrochemistry, Almaty 050010, Kazakhstan; a.naizabayev@ifce.kz (A.A.N.); f.buharbaeva@ifce.kz (F.U.B.); a.kenzheeva@ifce.kz (A.M.K.); a.auezkhanova@ifce.kz (A.S.A.); s.akhmetova@ifce.kz (S.N.A.); a.brodskiy@ifce.kz (A.R.B.); 2 Department of Physics and Technology, Al Farabi Kazakh National University, Almaty 050040, Kazakhstan; 3 Research Park, St. Petersburg University, St. Petersburg 199034, Russia; evgeniy.zhizhin@spbu.ru

**Keywords:** supported catalyst, palladium, titanium dioxide, magnetic nanoparticles, phenylacetylene hydrogenation

## Abstract

Recently, Pd catalysts supported on magnetic nanoparticles (MNPs) have attracted a great attention due to their ability of easy separation with an external magnet. Modification of MNPs is successfully used to obtain Pd magnetic catalysts with enhanced catalytic activity. In this work, we discussed the effect of titania content in TiO_2_/MNPs support materials on catalytic properties of Pd@TiO_2_/MNPs catalysts in phenylacetylene hydrogenation. TiO_2_/MNPs composites were prepared by simple ultrasound-assisted mixing of TiO_2_ and MNPs, synthesized by co-precipitation method. This was followed by deposition of palladium ions on the mixed metal oxides using NaOH as precipitant. The supports and catalysts were characterized using XRD, BET, STEM, EDX, XPS, and a SQUID magnetometer. Pd nanoparticles (5–6 nm) formed were found to be homogeneously distributed on support materials representing the well-mixed metal oxides with TiO_2_ content of 10, 30, 50, or 70%wt. Testing of the catalysts in phenylacetylene hydrogenation showed that their activity increased with increasing TiO_2_ content, and the process was faster in alkali medium (pH = 10). The hydrogenation rates of triple and double C–C bonds on Pd@70TiO_2_/MNPs achieved 9.3 × 10^−6^ mol/s and 23.1 × 10^−6^ mol/s, respectively, and selectivity to styrene was 96%. The catalyst can be easily recovered with an external magnet and reused for 12 runs without significant degradation in the catalytic activity. The improved catalytic properties of Pd@70TiO_2_/MNPs can be explained by the fact that the surface of the support is mainly composed of TiO_2_ particles, affecting the state and size of Pd species.

## 1. Introduction

Hydrogenation is one of the most important chemical processes widely used in the industrial production of specialty chemicals [1]. For instance, during fine chemical synthesis, various functional groups, such as –C≡C, –C=O, –NO_2_, –C≡N, –COOR, and –CONH_2_, can be selectively reduced by clean and cheap H_2_ to their corresponding alkenes, alcohols, and amines, which are key intermediates for fine chemicals, agrochemicals, polymers, and pharmaceuticals [2]. Among them, the selective hydrogenation of alkynes produced from petroleum have attracted considerable attention in both academia and industry for several decades [3]. More specifically, the removal of phenylacetylene from styrene stream, via such semi-hydrogenation, plays an important role in the styrene polymerization, as phenylacetylene impurity above a concentration of 10 ppm will poison the catalyst and reduce the quality of polystyrene product [4,5].

Heterogeneous catalysts using Pd nanoparticles as the main active components have been widely applied for selective hydrogenation due to their unique electronic structure and ability to adsorb and activate hydrogen and unsaturated substrates [2]. However, developing robust catalysts for alkyne semi-hydrogenation remains a major challenge [2,6].

Support material can play an important role in the design metal-supported catalysts with improved properties. Mesoporous materials with a high specific surface area provide the formation of highly dispersed forms of metal catalysts and prevent their agglomeration. Improving the dispersion of the metal catalyst on the support material usually increases their activity [7,8]. Interaction with the support material can also significantly affect the activity and selectivity of noble metal nanoparticles in various reactions including hydrogenation [9,10,11].

Various mesoporous materials such as carbon, alumina, silica, titanium dioxide, iron oxides, etc., are used as supports for metal catalysts [11]. Among them, magnetic iron oxide nanoparticles (MNPs) have recently become of particular interest due to their inherent unique properties such as: readily available, high surface area, high loading of active species immobilization, high degree of chemical stability in various organic and inorganic solvents and simple separation by an external magnet [12,13]. Remarkably, magnetic separation avoids filtration and centrifuge, which is very lucrative in large-scale industrial production and prevents loss of the catalyst during the recovery process [12,14].

In the last decade, a number of studies have been dedicated to design effective Pd magnetic catalysts for various reactions [15,16], including hydrogenation [17]. In some of them, magnetic support materials were modified in order to obtain supported Pd catalysts with improved properties. For example, in our prior work [18], Pd/MNPs’ catalyst modified with polyvinylpyrrolidone (PVP) and NaOH was studied in phenylacetylene hydrogenation. It was found that the NaOH adsorbed on magnetic support affects the size, stability, and catalytic properties of the Pd particles, and the effect of alkali is enhanced in the presence of the polymer, increasing the activity and lifetime of the catalyst. Rahimi E. et al. [19] reported that Fe_3_O_4_@Al_2_O_3_ proved to be an efficient support for palladium catalyst, and alumina coating had a considerable impact on the catalyst performance in reduction of nitrate from simulated groundwater. Kaur M. et al. [20] synthesized Pd nanoparticles decorated on ZnO/Fe_3_O_4_ cores and doped with Mn^2+^ and Mn^3+^ for catalytic C–C coupling, nitroaromatics reduction, and the oxidation of alcohols and hydrocarbons. The strong electronic interactions between Mn^2+^ and Pd make the Mn^2+^-doped nano-catalyst efficiently active and selective for reduction and oxidation. TiO_2_ is known as an excellent support for Pd hydrogenation catalyst [21,22]. However, Pd nanoparticles supported on Fe_3_O_4_/TiO_2_ magnetic composites have never been studied in the hydrogenation process.

In this study, we prepared Pd catalysts supported on TiO_2_/MNPs and examined their ability in the phenylacetylene hydrogenation. The catalysts were characterized using XRD, BET, STEM, EDX, XPS, and a SQUID magnetometer. The effect of titania content in mixed metal oxide support on properties of the catalysts was discussed.

## 2. Materials and Methods

### 2.1. Chemicals and Materials

FeCl_2_∙4H_2_O (98%, Sigma Aldrich, St. Louis, MO, USA), FeCl_3_∙6H_2_O (97%, Sigma Aldrich, St. Louis, MO, USA) NaOH (pure grade), TiO_2_ (anatase, 99.7%, Sigma Aldrich, St. Louis, MO, USA), PdCl_2_ (59–60% Pd, Sigma Aldrich, St. Louis, MO, USA), KCl (pure grade), methyl orange (pure, indicator grade, AppliChem, Darmstadt, Germany), ethanol (96.3%, pure grade) were used without additional purification. Phenylacetylene (98%, Sigma Aldrich, St. Louis, MO, USA) was purified by distillation and monitored by chromatography.

### 2.2. Synthesis of Magnetic Iron Oxide Nanoparticles (MNPs)

Magnetic iron oxide nanoparticles were synthesized by co-precipitation method according to the procedure [18,23] using sodium hydroxide as a precipitant. Briefly, a freshly prepared aqueous solution of iron salts (FeCl_3_∙6H_2_O—23.34 g and FeCl_2_∙4H_2_O—8.58 g in 300 mL) was placed in a thermostated round-bottom flask with three outlets, mixed and heated to 50 °C. Then, 100 mL of 3.45 M NaOH solution was added to the flask, stirred using bubbling nitrogen over a period of 4 h, and then cooled to room temperature. The resulting black precipitate was removed from the supernatant by magnetic separation, washed several times with DI water until neutral reaction of the wash water (pH = 6.6), and dried in air.

### 2.3. Preparation of TiO_2_/MNPs Magnetic Composites

TiO_2_/MNPs magnetic composites were prepared by suspending powders of TiO_2_ and MNPs in 50 mL of water using T-10 basic Ultra-Turrax homogenizer (IKA, Staufen, Germany). The mixtures were sonicated during 30 min at a frequency of 50–60 Hz, stirring rate of 14.500 rpm (rounds per minute) and ambient conditions. The resulting composites were removed from the supernatant by magnetic separation and dried in air. The amounts of TiO_2_ and MNPs were taken from calculation to obtain the composites, approximately 4 g of each, with TiO_2_ content of 10, 30, 50, or 70%wt., which were termed as 10TiO_2_/MNPs, 30TiO_2_/MNPs, 50TiO_2_/MNPs, and 70TiO_2_/MNPs, respectively.

### 2.4. Preparation of Palladium Catalysts

The catalysts were prepared by deposition of palladium (II) ions on the surface of TiO_2_/MNPs using NaOH as precipitant as follows. A 5 mL quantity of 1.9 × 10^−2^ M water solution of potassium (II) tetrachloropalladate (K_2_PdCl_4_) was added dropwise to the aqueous suspension of a support material (1 g TiO_2_/MNPs in 20 mL of water) and stirred for 2 h. Then, 2 mL of 0.25 M water solution of NaOH was added dropwise to the mixture and stirred for 1 h. Further, the resulting catalyst was removed from the supernatant by magnetic separation, washed several times with DI water until neutral reaction of the wash water, and dried in air. The completeness of Pd immobilization on support materials was assessed by the residual concentration of palladium ions in the supernatant after the deposition process. The supernatant solution was neutralized with 0.25 M of HCl in an amount equivalent to the added NaOH and analyzed to determine the concentration of [PdCl_4_]^2−^ ions. The palladium concentration in the supernatant solutions was determined on an SF-2000 UV/Vis spectrophotometer (OKB Spectr, Saint Petersburg, Russia) using calibration curves at a wavelength of λ = 430 nm. For a comparison, Pd@MNPs and Pd@TiO_2_ catalysts were prepared using the same procedure.

### 2.5. Characterization of the Composites and Catalysts

Powder X-ray diffraction (XRD) patterns were obtained with a DRON-4-0.7 X-ray diffractometer (Bourevestnik, Saint Petersburg, Russia) using cobalt-monochromatized Co Kα radiation (λ = 0.179 nm). Measurement of specific surface area was carried out by low-temperature N_2_ adsorption–desorption technique (BET) using Accusorb equipment (Micromeritics, Norcross, GA, USA). A 57Fe Mossbauer spectrum was recorded using an SM-2201 spectrometer at 293 K. The 57Co source used (~50 mCi) was in a Cr matrix. The elemental analysis was carried out using JSM-6610LV (JEOL, Tokyo, Japan) and Quanta 200i 3D (FEI Company, Hillsboro, OR, USA) scanning electron microscopes with EDX detectors. X-ray photoelectron spectra (XPS) of catalysts were recorded on an ESCALAB 250Xi X-ray and Ultraviolet Photoelectron spectrometer (Thermo Fisher Scientific, Waltham, MA, USA) with AlKα radiation (photon energy 1486.6 eV). Spectra were recorded in the constant pass energy mode at 50 eV for survey spectrum and 20 eV for element core level spectrum, using XPS spot size 650 μm. A total energy resolution of the experiment was about 0.3 eV. Investigations were carried out at room temperature in an ultrahigh vacuum of the order of 1 × 10^−9^ mbar. An ion-electronic charge compensation system was used to neutralize the sample charge. Magnetic properties of the magnetic materials were measured using an MPMS SQUID VSM DC Magnetometer (Quantum Design Inc., San Diego, CA, USA) at 300 K. High-angle annular dark-field scanning transmission electron microscopy (HAADF-STEM) micrographs were obtained on a Zeiss Libra 200FE transmission electron microscope (Carl Zeiss, Oberkochen, Germany) with an accelerating voltage of 100 kV.

### 2.6. Photocatalytic Properties of Support Materials

Photocatalytic activity of support materials was evaluated in the degradation of methyl orange under UV light at ambient conditions. The source of UV radiation was a quartz lamp with a wavelength of 254 nm and a power of 30 W. The process was carried out as follows. A 0.2 g amount of a photocatalyst (MNPs, TiO_2_, TiO_2_/MNPs) was stirred in 50 mL of DI water to obtain a homogeneous suspension. Then, 100 mL of 15 mg/mL methyl orange water solution was added to the suspension and stirred in the dark for 30 min to establish adsorption–desorption equilibrium. Further, the lamp was turned on, and the photodegradation of the dye was carried out. Aliquots of the dye (4 mL) were taken at certain intervals of time, centrifuged to remove a catalyst from the solution, and analyzed using an SF-2000 spectrophotometer at a wavelength of λ = 430 nm. The dye content in the aliquots was determined using calibration curves.

### 2.7. Phenylacetylene Hydrogenation

Hydrogenation was carried out in a thermostatically controlled long-necked glass flask reactor in an ethanol solution (25 mL) at 40 °C and atmospheric pressure of hydrogen, with intensive stirring (600–700 rpm). The catalyst (50 mg) was pre-treated with hydrogen directly in the reactor, and then 0.25 mL (2.23 mmol) of the phenylacetylene was introduced. The substrate amount was taken based on the uptake of 100 mL of hydrogen. The reaction rate was calculated as the hydrogen consumption per unit time. The reaction products were analyzed by gas chromatography on a Chromos GC-1000 chromatograph (Chromos, Dzerzhinsk, Russia) with a flame ionization detector using a BP21 (FFAP) capillary column with a polar phase (PEG modified with nitroterephthalate) of 50 m in length and a 0.32 mm inside diameter. The selectivity of the catalyst was calculated as the ratio of the target product to the sum of all reaction products at a fixed conversion. The catalysts were also tested in alkali medium (NaOH in ethanol, pH = 10) at the same reaction conditions. For the hydrogenation of phenylacetylene on Pd@70TiO_2_/MNPs, a variation in operating parameters such as the catalyst dosage (25–100 mg), amount of the substrate (0.25–1.00 mL), temperature (30–50 °C) was carried out. The reusability of the catalysts was evaluated by the hydrogenation rates in successive runs (0.25 mL, 2.23 mmol) using the same catalyst sample (50 mg) at 40 °C, pH = 10, and atmospheric pressure of hydrogen.

## 3. Results and Discussion

### 3.1. Characterization of Support Materials and Pd Catalysts

The crystallographic structure and composition of the MNPs synthesized by co-precipitation method were identified using XRD and Mossbauer spectroscopy. The TiO_2_/MNPs composites prepared by ultrasound-assisted mixing of the metal oxides were also characterized using the XRD method.

**Figure 1:**
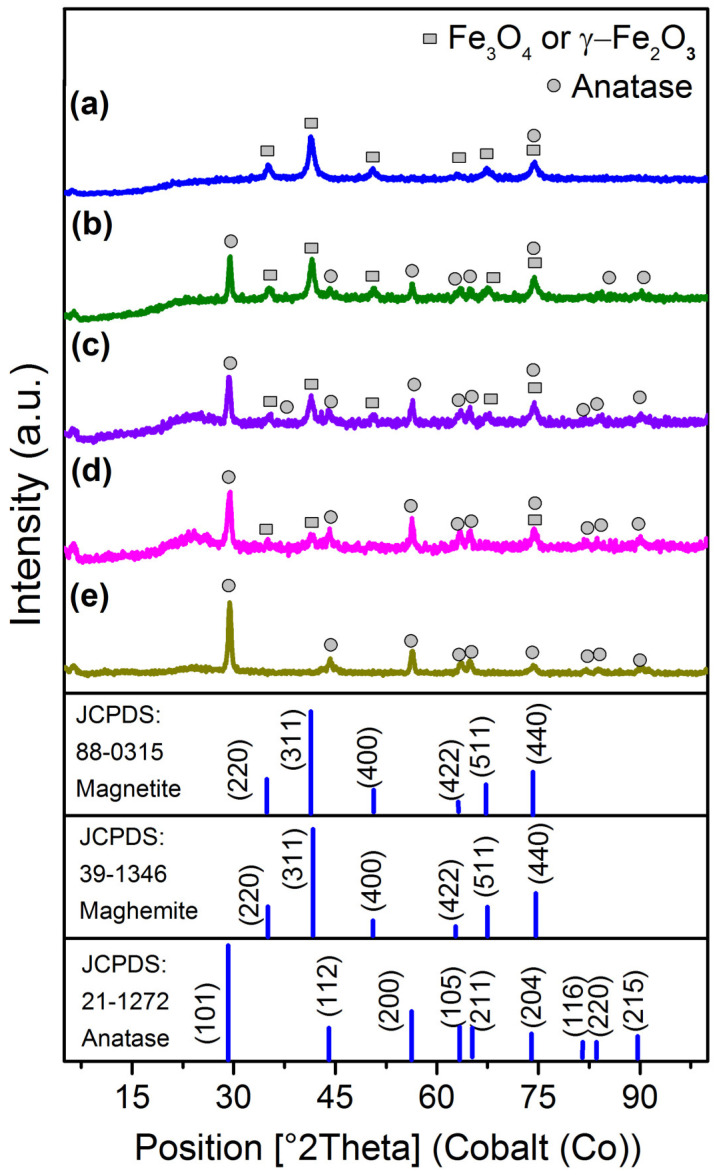
XRD patterns of MNPs (**a**), TiO_2_ (**e**), and TiO_2_/MNPs composites with TiO_2_ content of 70%wt. (**b**), 50%wt. (**c**), 30%wt. (**d**). According to XRD data, TiO_2_/MNPs composites contain TiO_2_ in anatase phase (circle) and an iron oxide in magnetite or maghemite phase (square).

Figure 1 shows the XRD patterns of the MNPs, TiO_2_, and TiO_2_/MNPs composites with different metal oxides’ ratio. The six characteristic peaks at 35.0°, 41.4°, 50.5°, 63.2°, 67.4°, and 74.4° corresponding to the (220), (311), (400), (422), (511), and (440) crystal planes, respectively, of magnetic iron oxides, Fe_3_O_4_ (JCPDS Card No. 88-0315) or γ-Fe_2_O_3_ (JCPDS Card No. 39-1346), with an inverse cubic spinel structure were observed in XRD patterns of the MNPs and TiO_2_/MNPs composites (Figure 1a–d) [24]. At the same time, the XRD patterns of TiO_2_/MNPs composites (Figure 1b–d) exhibited additional characteristic peaks at 29.3°, 44.0°, 56.3°, 63.4°, 64.9°, 74.4°, 81.8 °, 83.8°, and 90.1°, corresponding to the (101), (112), (200), (105), (211), (204), (116), (220), and (215) crystal planes, respectively, of the starting TiO_2_ (Figure 1e) in the anatase phase (JCPDS card No. 21-1272) [25]. Calculation with Scherrer’s formula for the strongest peaks reveals that the average crystallite sizes of the samples are 10.0 and 15.6 nm for the starting MNPs and TiO_2_, respectively [26]. The calculated sizes of MNPs and TiO_2_ particles in composites were near the same as in the starting materials and ranged from 8.7 to 11.6 nm and from 12.4 to 14.5 nm, respectively.

The isostructurality of Fe_3_O_4_ and γ-Fe_2_O_3_ and close values of crystal lattice parameters make it difficult to unambiguously identify diffractograms that correspond to magnetic powder particles [27]. For more accurate identification of the phase composition, the MNPs were analyzed using Mossbauer spectroscopy at 293 K.

**Figure 2:**
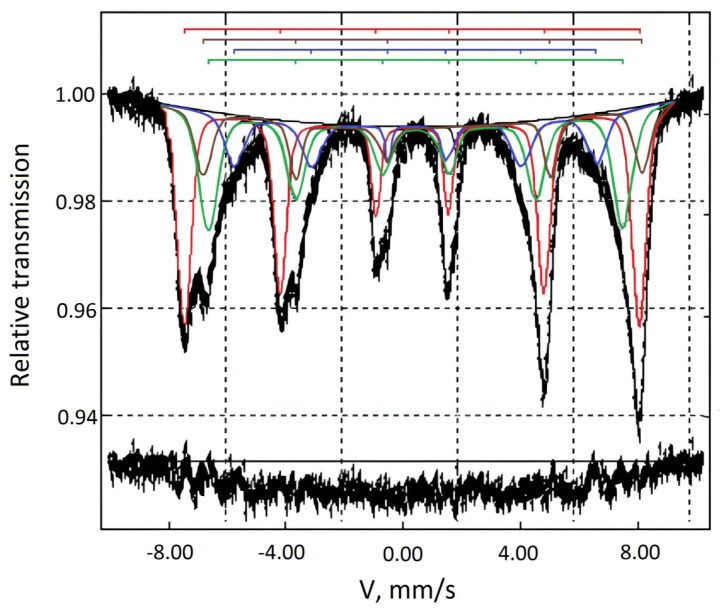
Mossbauer spectrum of MNPs. The spectrum contains four components (sextets) describing different local environments of a 57 Fe nuclide: sextet 1—Fe^3+^ in the A-positions (red line), sextet 2—Fe^2.5+^ in B-positions (blue line), sextet 3—Fe^3+^ in B-positions (green line), and sextet 4—Fe^3+^ in the surface regions (brown line).

The spectrum of starting MNPs is asymmetric and represents a superposition of four sextets (Figure 2), indicating the magnetically ordered state of the iron ions caused by agglomeration of the iron oxide nanoparticles [28].

The sextet parameters from the Mossbauer spectrum are presented in Table 1. The first sextet, having values of δ = 0.30 mm/s, ε = −0.01 mm/s, and H_n_ = 482 kOe, can be attributed to Fe^3+^ in the A-positions (tetrahedral sublattice) of both magnetite (Fe_3_O_4_) and maghemite (γ-Fe_2_O_3_) [29,30]. The second component (δ = 0.68 mm/s, ε = −0.01 mm/s and H_n_ = 465 kOe) is attributed to Fe^2.5+^ located on B-positions (octahedral sublattice) of the magnetite spinel structure (Fe_3_O_4_) [29]. The third sextet has values of δ = 0.43 mm/s, ε = −0.01 mm/s and H_n_ = 438 kOe, which are characteristic values for Fe^3+^ in B-positions (octahedral sublattice) of maghemite (γ-Fe_2_O_3_) [30]. The quadrupole splitting of −0.01 mm/s of the first three sextets is indicative of cubic symmetry [27]. The fourth component with values of δ = 0.44 mm/s, ε = −0.04 mm/s, and H_n_ = 383 kOe is probably attributed to iron ions located in the surface regions of γ-Fe_2_O_3_ nanoparticles, which are “depleted” in exchange bonds [30]. Thus, based on Mossbauer study, it can be concluded that the MNPs are composed of both magnetite and maghemite. The magnetite most likely is located inside and maghemite on the outside of the MNPs.

**Figure 3:**
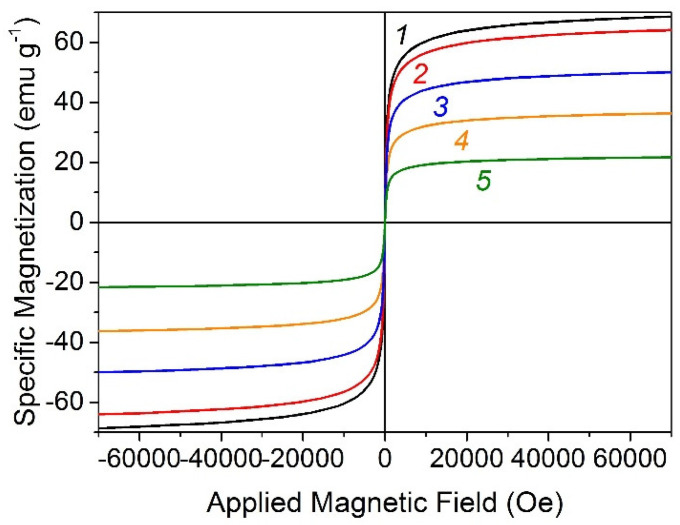
Magnetic hysteresis curves of MNPs (curve 1), 10TiO_2_/MNPs (curve 2), 30TiO_2_/MNPs (curve 3), 50TiO_2_/MNPs (curve 4), and 70TiO_2_/MNPs (curve 5) measured by SQUID magnetometer at room temperature.

The room-temperature magnetic hysteresis measurements of the MNPs and TiO_2_/MNPs composites were carried out at 300 K in the applied magnetic field, sweeping from −70 to 70 kOe. As shown in Figure 3 (curve 1), the specific magnetization at 20 kOe, remanent magnetization (M_r_), and coercivity (H_C_) of the starting sample of MNPs were found to be 63.6 emu/g, 1.83 emu/g, and 16 Oe, respectively. This suggests that the MNPs exhibit weak ferromagnetic and soft magnetic behaviors [31], which is consistent with Mossbauer spectroscopy data and indicates some agglomeration of MNPs [28]. The magnetization of the MNPs is lower than their bulk counterparts (86 and 76 emu/g for magnetite and maghemite at room temperature, respectively) and is not far from the value of 63–64 emu/g observed for the iron oxide nanoparticles of 8.3–9.4 nm in size [32]. The saturation magnetization (M_S_) of the TiO_2_/MNPs composites was decreased with decreasing the content of MNPs (Figure 3, curves 2–5).

The more detailed analysis of the hysteresis loops (Table 2) showed that the M_S_ and M_r_ values decreased proportionally to the expected content of MNPs in the composites. For example, a specific magnetization of 50TiO_2_/MNPs composite at 20 kOe was 34 emu/g or 53% from the value for starting MNPs. This suggests that metal oxides homogeneously distributed to each other and MNPs (or TiO_2_) content in the composites is close to calculated values of 10, 30, 50, and 70%wt.

At the same time, the coercivity of the composites has not significantly changed to compare with changes in M_S_ and M_r_ values for corresponding composites. Lee J.S. et al. [33] demonstrated that the coercivity of magnetic multi-granule nanoclusters composed of nearly the same size of granules (9.0–11.6 nm) can be decreased up to 97% (from 19.1 to 0.57 Oe) when the nanocluster diameter decreased from 53 to 32 nm. This suggests that the sizes of magnetic agglomerates were practically not changed after ultrasound-assisted mixing of MNPs with TiO_2_.

The results of nitrogen adsorption–desorption measurements were used to determine the specific surface area (S_BET_) of the MNPs, TiO_2_, and TiO_2_/MNPs composites. Specific surface area (S_XRD_) of the samples was also calculated using XRD data by the modified equation, proposed in [34]:

(1) $$S_{XRD}=\frac{6000a}{d(TiO_{2})\times 3.9}+\frac{6000b}{d(MNPs)\times 4.9},$$

where a and b—the content (from 0 to 1) of TiO_2_ and MNPs in a support material, respectively; d—average crystallite sizes of TiO_2_ and MNPs, calculated from XRD data using Scherrer’s formula; 3.9 and 4.9—density of TiO_2_ and MNPs.

A comparison of S_XRD_ and S_BET_ data from samples is presented in Table 3.

Values of a specific surface area calculated from XRD data were shown to be higher than those determined with the BET method. The positive values for the interface area among the particles, calculated using the formula (S_XRD_ – S_BET_)/2, indicate surface blocking of TiO_2_ and MNPs nanoparticles due to their agglomeration. The (S_XRD_ – S_BET_)/2 value for MNPs and TiO_2_/MNPs composites was higher compared with that for TiO_2_. This suggests that MNP-containing materials possess a higher agglomeration degree, and the surface of TiO_2_ particles can be blocked with MNPs after their mixing.

Relatively developed specific surface area of the resulting materials makes them good candidates for the preparation of supported Pd catalysts. It should be noted that all samples demonstrated nearly the same BET surface area, which allows to overlook the effect of surface area of a support material when comparing the behavior of the catalysts based on them in the hydrogenation process. In our prior work [35], it was found that TiO_2_ possesses a low affinity to [PdCl_4_]^2−^ ions. Therefore, in this study, Pd catalysts were prepared by deposition of [PdCl_4_]^2−^ ions on the support materials using sodium hydroxide.

Table 4 shows the results of assessing the degree of palladium ions’ deposition on MNPs, TiO_2_, and TiO_2_/MNPs composites.

According to photoelectric colorimetric (PEC) analysis, the addition of NaOH to a suspension containing [PdCl_4_]^2−^ and a support material promotes almost complete (95–99%) deposition of palladium ions onto the metal oxides (MNPs, TiO_2_, and TiO_2_/MNPs). The palladium content in the catalysts, calculated based on the PEC data, was about 1%, which is confirmed by elemental EDX analysis (Table 5). In addition, EDX elemental analysis data were used to calculate the content of TiO_2_ in the catalysts. For example, according to calculations, Pd@TiO_2_ was composed of 93% TiO_2_, 1% Pd, 6% moisture, and other (Al, Si, Cl, etc.) impurities. In the case of Pd@TiO_2_/MNPs catalysts, the TiO_2_ content was found to be close to calculated (expected) values of 10, 30, 50, and 70%wt. (Table 5), which is consistent with data obtained from the SQUID magnetometer (Table 2). It should be noted that Na was not detected in almost all catalysts. That is, the catalysts do not contain NaOH, which is able to affect the catalytic properties of the supported Pd nanoparticles [18].

**Figure 4:**
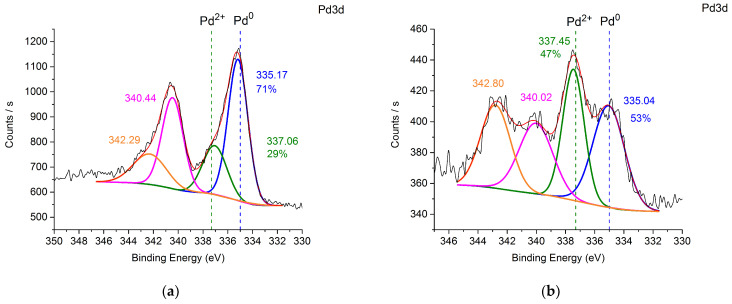

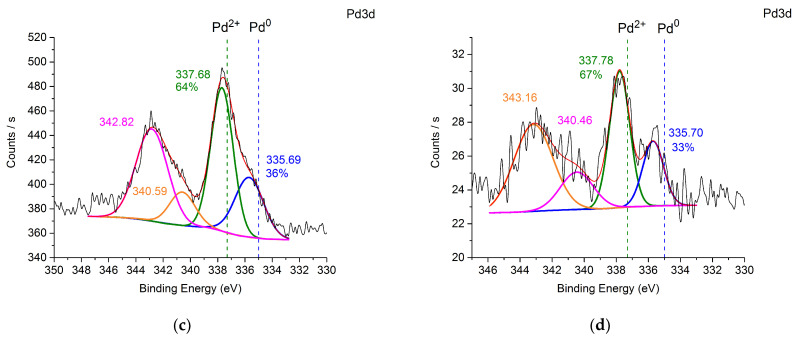
Pd 3d XPS spectra of Pd@TiO_2_ (**a**), Pd@70TiO_2_/MNPs (**b**), Pd@30TiO_2_/MNPs (**c**), and Pd@MNPs (**d**). The reference values of binding energies for Pd^0^ [36] and Pd^2+^ [37] are presented in spectra as dashed lines (green for Pd^2+^ and blue for Pd^0^).

In order to assess the state of palladium during the hydrogenation process, the catalysts were treated with hydrogen in the reactor at 40 °C and then studied using XPS. The deconvoluted Pd 3d signals (Figure 4) clearly illustrate the different oxidation states of Pd existing on the surface of the catalysts. In the Pd@TiO_2_ and Pd@70TiO_2_/MNPs, Pd 3d _5/2_ peaks with binding energies at around 335 eV and 337 eV can be attributed to Pd in 0 and +2 oxidation states, respectively (Figure 4a,b) [36,37], where the metallic Pd was the dominant species (>50%). On contrary, in samples Pd@30TiO_2_/MNPs and Pd@MNPs (Figure 4c,d), palladium was mostly in oxidized form. Moreover, the binding energy of Pd^0^ and Pd^2+^ have a positive shift (ca. 0.4–0.7 eV) [37], probably due to electron transfer from Pd species to iron oxide [38] (Table 6).

**Figure 5:**
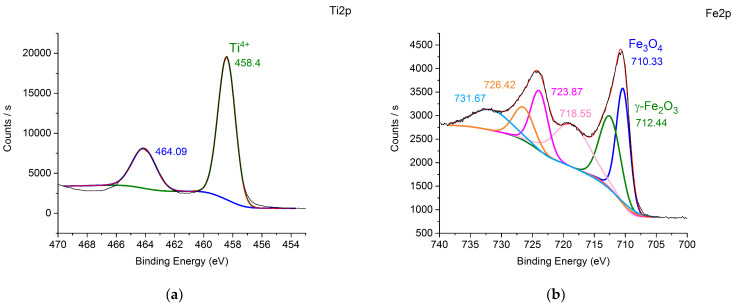

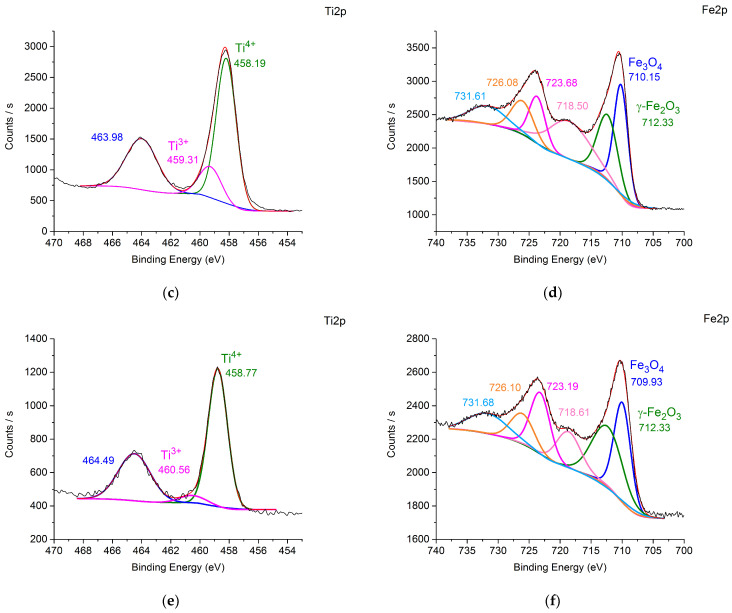
Ti 2p (**a**,**c**,**e**) and Fe 2p (**b**,**d**,**f**) XPS spectra of Pd@TiO_2_ (**a**), Pd@MNPs (**b**), Pd@30TiO_2_/MNPs (**d**,**e**), and Pd@70TiO_2_/MNPs (**c**,**f**).

Figure 5 shows Ti 2p and Fe 2p regions of the XPS spectra of the Pd@TiO_2_, Pd@70TiO_2_/MNPs, Pd@30TiO_2_/MNPs, and Pd@MNPs catalysts. The two strong peaks from the Pd@TiO_2_ at around 464.1 eV and 458.4 eV with symmetry can be attributed to Ti 2p _1/2_ and Ti 2p _3/2_, respectively (Figure 5a). The peak positions and 5.7 eV peak separation of the Ti 2p doublet agree well with the energy reported for TiO_2_ nanoparticles [39]. The Ti 2p regions of the XPS spectra of the Pd@70TiO_2_/MNPs and Pd@30TiO_2_/MNPs (Figure 5c,e) were nearly the same as for Pd@TiO_2_, except that small shoulders at around 460 eV were observed, indicating the presence of the Ti^3+^ in both samples, probably due to an interaction of TiO_2_ with MNPs [40] (Table 6).

The peaks from the Pd@70TiO_2_/MNPs, Pd@30TiO_2_/MNPs, and Pd@MNPs (Figure 5b,d,f) at around 710 eV and 712 eV can be fitted with two configurations of Fe 2p _3/2_, which can be ascribed to Fe_3_O_4_ and γ-Fe_2_O_3_, respectively. The weak peak at 719 eV is the Fe^3+^ shake satellite peak of the γ-Fe_2_O_3_ [41]. Thus, the Fe 2p XPS spectra confirm that MNPs are composed of both Fe_3_O_4_ and γ-Fe_2_O_3_. The presence of Fe^2+^ of Fe_3_O_4_ on the catalysts’ surface can also be caused by electron transfer from Pd species to MNPs [38]. In addition, the binding energy of the Fe 2p _3/2_ peak at around 710 eV for Pd@TiO_2_/MNPs catalysts was shifted towards smaller energies compared with that for Pd@MNPs (ca. 0.1–0.4 eV), confirming that the interaction between TiO_2_ and MNPs took place.

**Table 6:** Results of XPS analysis of the catalysts.

Region	Catalyst	Experimental BE Values, eV	BE Values in Literature, eV	Proposed Metal State	Ref.
Pd3d	Pd@TiO_2_	335.2	335.0	Pd^0^	[36]
		337.1	337.3	Pd^2+^	[37]
	Pd@70TiO_2_/MNPs	335.0	335.0	Pd^0^	[36]
		337.5	337.3	Pd^2+^	[37]
	Pd@30TiO_2_/MNPs	335.7	335.0	Pd^0^	[36]
		337.7	337.3	Pd^2+^	[37]
	Pd@MNPs	335.7	335.0	Pd^0^	[36]
		337.8	337.3	Pd^2+^	[37]
Ti2p	Pd@TiO_2_	458.4	458.6	Ti^4+^	[40]
	Pd@70TiO_2_/MNPs	458.2	458.6	Ti^4+^	[40]
		459.3	460.2	Ti^3+^	
	Pd@30TiO_2_/MNPs	458.8	458.6	Ti^4+^	[40]
		460.6	460.2	Ti^3+^	
Fe2p	Pd@70TiO_2_/MNPs	709.9	708.5	Fe_3_O_4_	[41]
		712.3	711.0	γ-Fe_2_O_3_	
	Pd@30TiO_2_/MNPs	710.2	708.5	Fe_3_O_4_	[41]
		712.3	711.0	γ-Fe_2_O_3_	
	Pd@MNPs	710.3	708.5	Fe_3_O_4_	[41]
		712.4	711.0	γ-Fe_2_O_3_	

**Figure 6:**
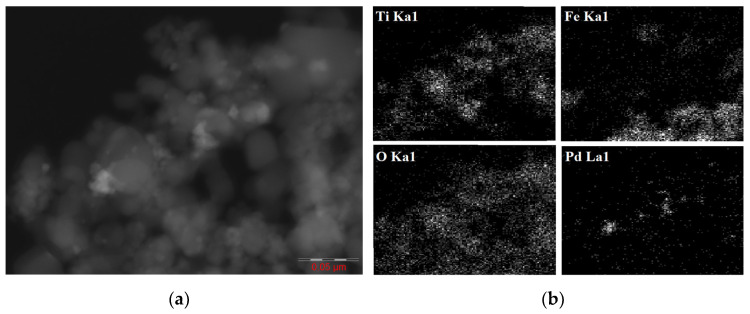
HAADF-STEM microphotograph (**a**) and EDX elemental mapping images (**b**) of Ti, Fe, O, and Pd from the Pd@70TiO_2_/MNPs catalyst.

Figure 6 shows an HAADF-STEM microphotograph and EDX elemental mapping images of Ti, Fe, O, and Pd from the Pd@70TiO_2_/MNPs treated with hydrogen, according to which the catalyst was composed of three phases. The largest particles (15–50 nm), forming an aggregate with clearly traced boundaries, correspond to titanium dioxide. Some sites of this TiO_2_ aggregate are covered with more compact aggregates composed of smaller MNPs (8–12 nm). The smallest particles observed on the surface of both TiO_2_ and MNPs aggregates belong to Pd^0^ (the brightest spots in the HAADF-STEM image) and PdO species. Of note, HAADF-STEM data are consistent with the results of XRD and BET studies, confirming the size and agglomeration of MNPs and TiO_2_ particles.

**Figure 7:**
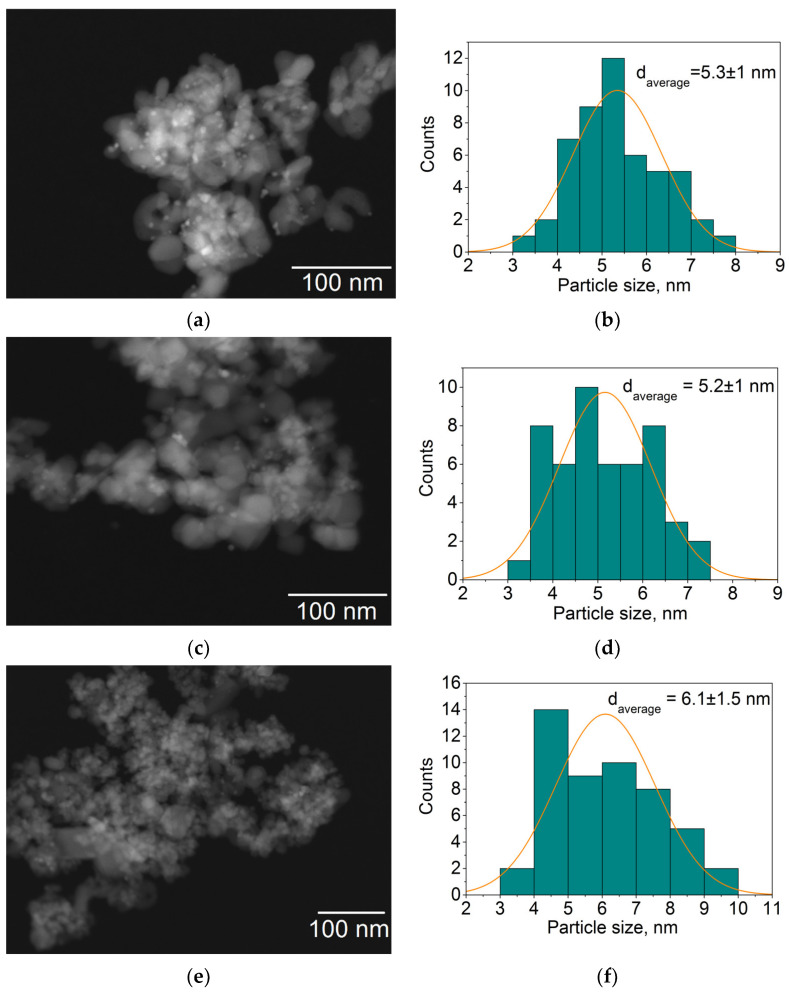
HAADF-STEM images (**a**,**c**,**e**) and corresponding Pd particle size distribution histograms (**b**,**d**,**f**) of the Pd@TiO_2_ (**a**,**b**), Pd@70TiO_2_/MNPs (**c**,**d**), and Pd@30TiO_2_/MNPs (**e**,**f**) catalysts.

A comparison of HAADF-STEM microphotographs obtained at lower magnification from the Pd@TiO_2_, Pd@70TiO_2_/MNPs, and Pd@30TiO_2_/MNPs catalysts (Figure 7a,c,e) shows that, in all cases, small spherical Pd nanoparticles (5–6 nm) are uniformly distributed on the surface of the supports. It should be noted that the surface of TiO_2_ aggregates in Pd@30TiO_2_/MNPs was more significantly blocked with MNPs compared with that of Pd@70TiO_2_/MNPs. Consequently, in the case of Pd@30TiO_2_/MNPs, the Pd particles mainly located on the surface of MNPs, while a predomination of palladium on the surface of TiO_2_ particles was observed for Pd@70TiO_2_/MNPs, which is consistent with results of XPS studies. In addition, Pd particles in Pd@TiO_2_ (5.3 nm) and Pd@70TiO_2_/MNPs (5.2 nm) were slightly smaller than those in the Pd@30TiO_2_/MNPs (6.1 nm) catalyst (Figure 7b,d,f).

### 3.2. Photocatalytic Properties of the Support Materials

TiO_2_ is an excellent support for Pd catalysts due to its strong metal–support interaction, chemical stability, and suitable acid-base properties [10,42]. In the case of mixed oxides of MNPs and TiO_2_, the catalytic properties of supported catalysts can be depended on an availability of TiO_2_ for Pd particles. TiO_2_ is well known as an efficient photocatalyst due to its excellent optical and electronic properties [43]. Therefore, the presence of TiO_2_ on the surface of TiO_2_/MNPs composites can be assessed by testing their photocatalytic properties.

**Figure 8:**
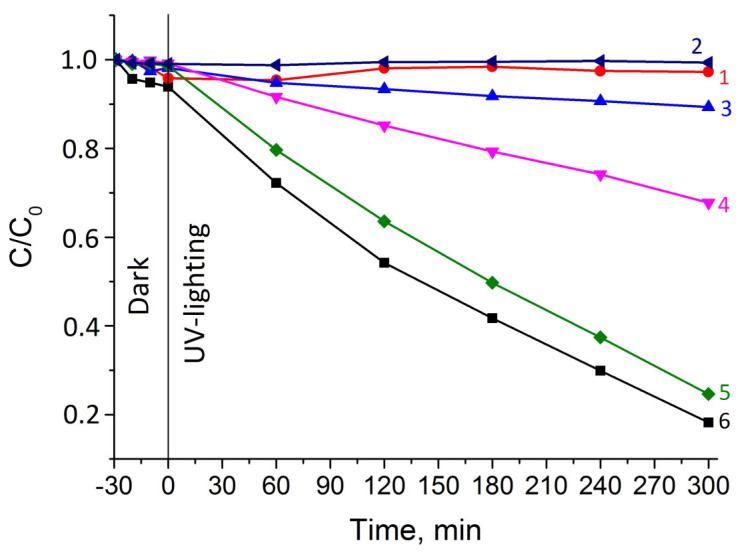
MO degradation curves in the presence of MNPs (curve 1), 10TiO_2_/MNPs (curve 2), 30TiO_2_/MNPs (curve 3), 50TiO_2_/MNPs (curve 4), 70TiO_2_/MNPs (curve 5), and TiO_2_ (curve 6). Reaction conditions: 0.2 g of a catalyst; initial MO concentration—10 mg/L; the volume of a solution—150 mL; wavelength—254 nm; lamp power—30 W.

The photocatalytic properties of MNPs, TiO_2_, and TiO_2_/MNPs were tested in the degradation of methyl orange (MO) under UV radiation (Figure 8).

The test results showed that MNPs and 10TiO_2_/MNPs do not possess photocatalytic activity. However, further increasing the TiO_2_ content in the composites leads to improving their photocatalytic properties. The removal of MO dye was 10%, 32%, and 75% for 30TiO_2_/MNPs, 50TiO_2_/MNPs, and 70TiO_2_/MNPs composites, respectively. It should be noted that 70TiO_2_/MNPs demonstrated a near-same activity as a starting TiO_2_ (82% of MO removed). The photodegradation rate, calculated from the dye removal data, was found to be 0.46 × 10^−10^, 1.08 × 10^−10^, 2.51 × 10^−10^, and 2.76 × 10^−10^ for 30TiO_2_/MNPs, 50TiO_2_/MNPs, 70TiO_2_/MNPs, and TiO_2_, respectively. The results obtained suggest that the presence of TiO_2_ on the surface of TiO_2_/MNPs can be achieved when its content in the composite is higher than 30%wt. Among the composites, the 70TiO_2_/MNPs were shown to possess the highest concentration of TiO_2_ particles on their surface comparable with starting TiO_2_, which is consistent with the results of HAADF-STEM studies of Pd@TiO_2_ and Pd@70TiO_2_/MNPs catalysts.

### 3.3. Phenylacetylene Hydrogenation

**Figure 9:**
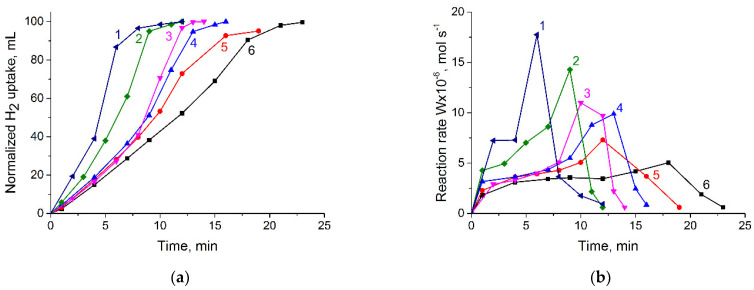
Variation in the hydrogen uptake (**a**) and rate of the reaction (**b**) versus time during the hydrogenation of phenylacetylene in the presence of Pd@TiO_2_ (curve 1); Pd@70TiO_2_/MNPs (curve 2); Pd@50TiO_2_/MNPs (curve 3); Pd@30TiO_2_/MNPs (curve 4); Pd@10TiO_2_/MNPs (curve 5); and Pd@MNPs (curve 6). Reaction conditions in text.

The catalytic performance of Pd catalysts supported on MNPs, TiO_2_, and TiO_2_/MNPs was evaluated in the selective hydrogenation of phenylacetylene. The hydrogenation experiments were carried out in ethanol at 0.1 MPa H_2_ and 40 °C. Figure 9a shows the variation in H_2_ uptake versus time during the hydrogenation process. The Pd@TiO_2_ and Pd@70TiO_2_/MNPs demonstrated the higher catalytic activity, reaching the semi-hydrogenation point (50 mL) after 4 and 6 min, respectively (Figure 9a, curves 1 and 2). The catalytic activity of the rest of the catalysts was lower and reached the semi-hydrogenation point after 8, 9, 10, and 12 min for Pd@50TiO_2_/MNPs, Pd@30TiO_2_/MNPs, Pd@10TiO_2_/MNPs, and Pd@MNPs, respectively (Figure 9a, curves 3, 4, 5, and 6). It should be noted that in the initial period, the hydrogen uptake for Pd@50TiO_2_/MNPs, Pd@30TiO_2_/MNPs, and Pd@10TiO_2_/MNPs was nearly the same. However, after reaching a semi-hydrogenation point, the rate of hydrogen uptake was different (Figure 9a, curves 3, 4, and 5). The phenylacetylene hydrogenation rate, calculated from the hydrogen uptake data, is presented in Figure 9b. In all cases, the reaction rate increased in the first two minutes and remained constant until a semi-hydrogenation point. Then, the reaction rate increased and, after passing a maximum, sharply decreased. The increasing of TiO_2_ content in the catalysts was accompanied by increasing of the maximum rate, observed after reaching the semi-hydrogenation point (Figure 9b).

**Figure 10:**
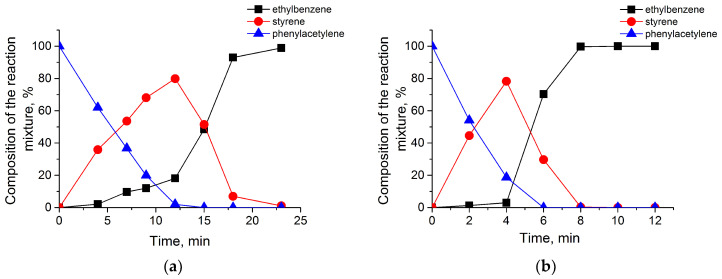

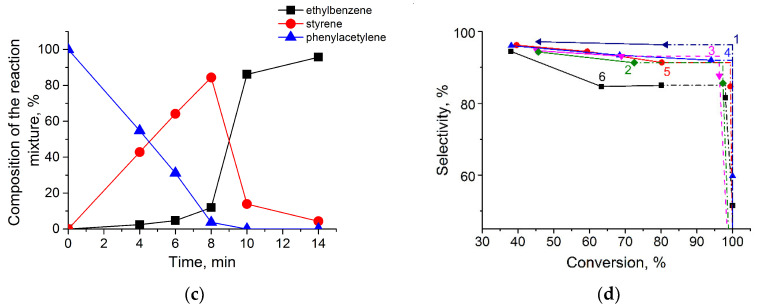
The results of chromatographic analysis: changes in the composition of the reaction mixture during the hydrogenation of phenylacetylene in the presence of Pd@MNPs (**a**), Pd@TiO_2_ (**b**), and Pd@50TiO_2_/MNPs (**c**); dependence of selectivity to styrene with the substrate conversion (**d**) for Pd@TiO_2_ (curve 1), Pd@70TiO_2_/MNPs (curve 2), Pd@50TiO_2_/MNPs (curve 3), Pd@30TiO_2_/MNPs (curve 4), Pd@10TiO_2_/MNPs (curve 5), and Pd@MNPs (curve 6). Reaction conditions in text.

According to the chromatographic analysis, styrene is accumulated on Pd@MNPs, Pd@TiO_2_, and Pd@50TiO_2_/MNPs in the initial period and then is reduced to ethylbenzene (Figure 10a–c). Accumulation of styrene was accompanied by the formation of a small amount of ethylbenzene, and its yield at the semi-hydrogenation point for Pd@MNPs (17.5%) was higher than that for Pd@TiO_2_ (3%) and Pd@50TiO_2_/MNPs (12%) catalysts. At the same time, the maximum yield of styrene was close to 80% for all catalysts. The composition of the reaction mixture was changed similarly during phenylacetylene hydrogenation on the rest of the Pd@TiO_2_/MNPs catalysts. Dependence of selectivity on conversion for all catalysts tested shows that the titania-containing catalysts possessed a better selectivity to styrene to compare with Pd@MNPs (Figure 10d).

The hydrogenation rate and selectivity to styrene were calculated from the hydrogen uptake and chromatographic analysis data, respectively. A comparison of the catalytic properties of the catalysts during the hydrogenation of phenylacetylene is presented in Table 7.

Accumulation of styrene on Pd@MNPs occurred selectively (85%) at a rate of 3.5 × 10^–6^ mol/s, and then the rate increased to 5.3 × 10^–6^ mol/s, corresponding to hydrogenation of double C–C bond. The W_C≡C_ to W_C=C_ rates ratio was 1:1.5. The Pd@TiO_2_/MNPs magnetic catalysts showed improved catalytic properties, and their activity increased with increasing the titania content. Hydrogenation rates of triple and double C–C bonds on Pd@70TiO_2_/MNPs reached 7.0 × 10^–6^ and 14.3 × 10^–6^ mol/s, respectively, which are close to W_C≡C_ and W_C=C_ values observed for Pd@TiO_2_. The W_C≡C_ to W_C=C_ rates ratio was also changed to 1:2. However, the selectivity to styrene on the Pd@TiO_2_/MNPs and Pd@TiO_2_ catalysts was higher than 90%.

**Figure 11:**
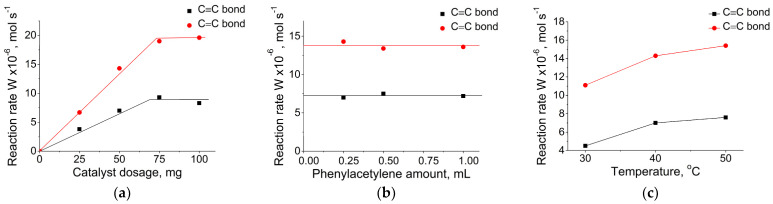
Effect of the variation in reaction parameters on activity of Pd@70TiO_2_/MNPs catalyst in the phenylacetylene hydrogenation: catalyst dosage (**a**); phenylacetylene amount (**b**); temperature (**c**). Reaction conditions: 40 °C, 0.1 MPa, catalyst 25–100 mg, phenylacetylene 0.25 mL, ethanol 25 mL (**a**); 40 °C, 0.1 MPa, catalyst 50 mg, phenylacetylene 0.25–1.00 mL, ethanol 25 mL (**b**); 30–50 °C, 0.1 MPa, catalyst 50 mg, phenylacetylene 0.25 mL, ethanol 25 mL (**c**).

To determine optimal reaction conditions, a series of experiments on investigation of the kinetics of phenylacetylene hydrogenation over Pd@70TiO_2_/MNPs were performed. The reaction parameters such as catalyst dosage (25–100 mg), phenylacetylene amount (0.25–1.00 mL), and temperature (30–50 °C) were varied (Figure 11). Figure 11a shows that the reaction rates (W_C≡C_ and W_C=C_) are proportional to the amount of the catalyst in the range of 25–75 mg. The rates of reaction increased linearly with increasing the catalyst amount, which accompanied with change in the W_C≡C_ to W_C=C_ rates ratio. A further increase in catalyst amount (100 mg) did not affect the rates of reaction. This result also suggests that measurements under the experimental conditions (50 mg of the catalyst) studied are within the kinetic regime. A variation in the phenylacetylene amount did not affect the rate (Figure 11b), and the reaction seemed to be of zero order to phenylacetylene under the reaction conditions studied. Increasing the reaction temperature from 30 to 40 °C led to an increase in the hydrogenation rates, while further increase in temperature did not significantly affect the efficiency of the process (Figure 11c). Thus, based on the results obtained, further catalytic studies were carried out at the following conditions: 50 mg of a catalyst, 0.25 mL of phenylacetylene at 40 °C.

**Figure 12:**
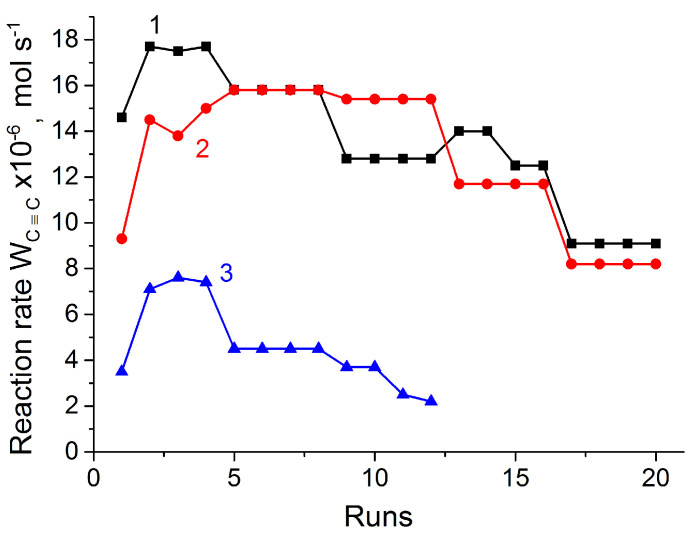
Reuse of Pd@TiO_2_ (curve 1), Pd@70TiO_2_/MNPs (curve 2), and Pd@MNPs (curve 3) catalysts. Reaction conditions: 50 mg of catalyst, 0.25 mL of phenylacetylene, 25 mL of ethanol, pH = 10, at 40 °C and 0.1 MPa.

In our prior study [18], it was shown that the modification of the surface of Pd magnetic catalysts with NaOH led to significant increase in their activity and lifetime. Such behavior can be explained by the effect of pH-dependent surface charging of metal oxides [44]. Therefore, in this study, the catalysts obtained were also tested in alkali medium. For this purpose, the solvent (ethanol) was adjusted with NaOH to pH = 10 and then used in the hydrogenation process. The results obtained (Table 7) show the increased activity of the catalysts in alkali medium. Hydrogenation rates of double C–C bonds increased up to 9.8 × 10^–6^ and 8.9 × 10^–6^ mol/s, while W_C≡C_ did not increase on Pd@MNPs and Pd@10TiO_2_/MNPs catalysts, respectively. It is worth noting that both catalysts demonstrated nearly the same catalytic properties (activity and selectivity). This is consistent with photocatalytic studies data, according to which it was proposed that the surface of 10TiO_2_/MNPs composite does not contain available TiO_2_ sites. In the case of the rest of the catalysts, increasing both the W_C≡C_ and W_C=C_ rates was observed. The W_C≡C_ to W_C=C_ rates ratio was also changed to 1:3 for the catalysts with higher MNP content (more than 50%wt.), while this value for Pd@70TiO_2_/MNPs and Pd@TiO_2_ almost remained unchanged. The selectivity to styrene achieved 95–96% for the catalysts containing more than 30%wt. of titania. The Pd@70TiO_2_/MNPs was found to be the most optimal catalyst due to the combination of excellent catalytic properties of Pd@TiO_2_ and magnetic properties of Pd@MNPs. The catalyst was recovered with an external magnet and then reused during 12 runs without loss in its activity (Figure 12, curve 2). The Pd@TiO_2_ demonstrated near-same activity during reuse (Figure 12, curve 1), while the W_C≡C_ hydrogenation rate on Pd@MNPs was significantly lower and gradually decreased after 8 runs (Figure 12, curve 3).

Thus, the Pd@70TiO_2_/MNPs catalyst demonstrated improved catalytic properties in alkali medium. The values of selectivity to styrene (96%), the reaction rate in terms of TOF (2.0 s^−1^), and stability in terms of TON (19,400) were comparable with those indicated for other known Pd catalysts supported on magnetic core–shell composites (Table 8). Moreover, after the 1st run, the activity of the catalyst increased, and the reaction rate in terms of TOF was found to be up to 3.4 s^−1^ for the next 11 runs. Further, the reaction rate decreased, and the TOF value for the 20th run was 1.7 s^−1^, which was close to that for the 1st run (Figure 12).

## 4. Conclusions

In summary, we have successfully prepared well-mixed iron and titanium oxides (TiO_2_/MNPs composites) followed by deposition of [PdCl_4_]^2−^ ions on their surface to obtain supported Pd magnetic catalysts. For comparison, Pd@TiO_2_ and Pd@MNPs catalysts were also prepared. The catalysts were studied in phenylacetylene hydrogenation. The main aim was to gain insights into the effect of titania content in mixed metal oxide support on properties of the catalysts. A comparative study revealed some noticeable facts as the phenylacetylene hydrogenation rates were higher in alkali medium (pH = 10), the activity of Pd@TiO_2_/MNPs catalysts increased with increasing the TiO_2_ content, and the Pd@70TiO_2_/MNPs was found to be the most optimal catalyst due to combination of excellent catalytic properties of Pd@TiO_2_ (activity, selectivity, and stability) and magnetic properties of Pd@MNPs. This can be explained by the fact that the surface of 70TiO_2_/MNPs mainly composes of TiO_2_ particles, and, therefore, they are more available for Pd nanoparticles compared with the rest of the TiO_2_/MNPs composites. This was confirmed by testing of TiO_2_/MNPs composites in photodegradation of MO dye. The results showed that their photocatalytic activity increased with increasing the TiO_2_ content in composites, and 70TiO_2_/MNPs demonstrated near-same photocatalytic properties as a starting TiO_2_. In addition, according to XPS study, the palladium lines from Pd@TiO_2_ and Pd@70TiO_2_/MNPs were not shifted towards higher energies, while for catalysts with higher MNPs content, some positive shift was observed. It should also be noted that Pd nanoparticles supported on TiO_2_ and 70TiO_2_/MNPs were nearly the same in size (5 nm) and slightly smaller compared with those of Pd@30TiO_2_/MNPs (6 nm). This suggests that composition of surface layer of the two-component TiO_2_/MNPs composites affects the state of Pd species supported on them and, therefore, their behavior in the hydrogenation process. All in all, the composites, containing TiO_2_ and magnetic iron oxides, were shown to be promising for the development of Pd hydrogenation catalysts. We believe that the approach on evaluation of photocatalytic properties of hybrid supports, firstly used in this work, will be useful in the development of such types of catalysts.

## Figures and Tables

**Table 1:** Room-temperature Mossbauer parameters of MNPs. δ is the isomer shift relative to α-Fe, ε is the quadruple splitting, H_n_ is the magnetic hyperfine field at Fe nuclei, I is the relative occupancy of the position (VI—octahedral and IV—tetrahedral).

Sextet Number	δ, mm/s	ε, mm/s	H_n_, kOe	I, %	Position/Phase
1	0.30	−0.01	482	41	Fe^3+^—IV/Fe_3_O_4_ and γ-Fe_2_O_3_
2	0.68	−0.01	465	13	Fe^2.5+^—VI/Fe_3_O_4_
3	0.43	−0.01	438	30	Fe^3+^—VI/γ-Fe_2_O_3_
4	0.44	−0.04	383	16	Fe^3+^ in surface of γ-Fe_2_O_3_

**Table 2:** The data on magnetic properties of samples extracted from hysteresis loops.

Samples	Magnetization at 20 kOe, emu/g	M_r_, emu/g	H_C_, Oe	Calculated Content of the MNPs in Magnetic Samples, %
MNPs	63.6	1.83	16.0	100%
10TiO_2_/MNPs	60.0	1.56	15.0	94.3%
30TiO_2_/MNPs	46.8	1.27	15.0	73.6%
50TiO_2_/MNPs	34.0	0.95	14.0	53.5%
70TiO_2_/MNPs	20.3	0.57	12.0	31.9%

**Table 3:** Results of study of support materials using XRD and BET.

Samples	Crystalline Size (XRD), nm		Specific Surface Area (S), m^2^/g		(S_XRD_ − S_BET_)/2, m^2^/g
	TiO_2_	MNPs	XRD	BET	
MNPs	–	10.0	122.4	74.3	24.1
30TiO_2_/MNPs	14.5	8.9	128.1	73.4	27.4
50TiO_2_/MNPs	13.0	8.7	129.5	72.2	28.7
70TiO_2_/MNPs	12.4	11.6	118.5	68.5	25.0
TiO_2_	15.6	–	98.6	65.9	16.4

**Table 4:** Results of assessing the degree of deposition of palladium ions on support materials.

Catalyst	Amount of Pd in Mother Liquor, ×10^−5^ mol		The Degree of Deposition, %	Pd Content in a Catalyst (calc.), %
	Before Deposition	After Deposition		
Pd@MNPs	9.50	0.08	99.2	0.99
Pd@10TiO_2_/MNPs	9.50	0.47	95.0	0.95
Pd@30TiO_2_/MNPs	9.50	0.38	96.0	0.96
Pd@50TiO_2_/MNPs	9.50	0.25	97.4	0.97
Pd@70TiO_2_/MNPs	9.50	0.19	98.0	0.98
Pd@TiO_2_	9.50	0.13	98.6	0.99

**Table 5:** Results of EDX elemental analysis of the Pd@MNPs, Pd@TiO_2_, and Pd@TiO_2_/MNPs catalysts.

Catalyst	Element Content, %wt.						Calc-d TiO_2_ Content, %
	Fe	Ti	O	Pd	Na	Al, Si, Cl, etc.	
Pd@MNPs	70.50	–	27.64	1.21	–	0.65	–
Pd@10TiO_2_/MNPs	61.17	5.40	31.54	1.05	0.12	0.72	9.02
Pd@30TiO_2_/MNPs	49.77	17.50	31.48	1.01	–	0.24	29.22
Pd@50TiO_2_/MNPs	36.04	28.46	33.86	1.39	–	0.25	47.51
Pd@70TiO_2_/MNPs	22.38	39.88	36.06	1.43	–	0.25	66.58
Pd@TiO_2_	–	55.79	41.13	1.03	–	2.05	93.14

**Table 7:** A comparison of catalytic properties of Pd@MNPs, Pd@TiO_2_, and Pd@TiO_2_/MNPs catalysts in phenylacetylene hydrogenation.

Catalyst	W × 10^−6^, mol/s		The W_C≡C_ to W_C=C_ Rates Ratio	Selectivity, %	Conversion, %
	C≡C	C=C			
At normal pH					
Pd@MNPs	3.5	5.3	1:1.5	85	80
Pd@10TiO_2_/MNPs	4.3	7.3	1:1.7	91	80
Pd@30TiO_2_/MNPs	4.4	9.9	1:2.3	92	94
Pd@50TiO_2_/MNPs	5.1	11.0	1:2.2	93	69
Pd@70TiO_2_/MNPs	7.0	14.3	1:2.0	92	79
Pd@TiO_2_	7.3	17.8	1:2.4	96	81
At pH = 10					
Pd@MNPs	3.5	9.8	1:2.8	88	91
Pd@10TiO_2_/MNPs	3.1	8.9	1:2.9	87	85
Pd@30TiO_2_/MNPs	5.1	15.1	1:3.0	95	44
Pd@50TiO_2_/MNPs	6.6	21.3	1:3.2	96	43
Pd@70TiO_2_/MNPs	9.3	23.1	1:2.5	96	53
Pd@TiO_2_	14.8	26.3	1:1.8	95	50

Reaction conditions: 50 mg of catalyst, 0.25 mL of phenylacetylene, 25 mL of ethanol, at 40 °C and 0.1MPa.

**Table 8:** A comparison of catalytic properties of the Pd@70TiO_2_/MNPs with those of other Pd magnetic catalysts.

Catalyst	Method of Preparation	Reaction Conditions	TOF ^a^, s^−1^	S ^b^, %	TON	Ref.
Pd@70TiO_2_/MNPs	Precipitation of Pd^2+^ ions on mixture of TiO_2_ and MNPs	50 mg of catalyst, 2.3 mmol of phenylacetylene in ethanol (pH = 10) at 40 °C and 0.1 MPa H_2_	2.0	96	19,400	This study
Pd-PVP/MNPs	Adsorption of Pd^2+^ ions on PVP-modified MNPs obtained using 1.5-fold excess of NaOH	50 mg of catalyst, 2.3 mmol of phenylacetylene in ethanol at 40 °C and 0.1 MPa H_2_	0.9	94	56,000 ^c^	[18]
Pd/Ni@G	Precipitation of Pd^2+^ ions on magnetic Ni@G core–shell particles	25 mg of catalyst, 1.85 mmol of phenylacetylene in ethanol at 30 °C and 0.2 MPa H_2_	2.0	93	8000	[45]
Fe_3_O_4_@ZIF-8/Pd	Precipitation of Pd^2+^ ions on Fe_3_O_4_@ZIF-8 core–shell composite	150 mg of catalyst, 49 mmol of phenylacetylene in methanol at 40 °C and 0.1 MPa H_2_	0.5	93	34,700	[46]

^a^ For hydrogenation of triple C–C bond; ^b^ Selectivity towards styrene; ^c^ Pd-PVP/MNPs was reused till its deactivation.

## Data Availability

The data that support the findings of this study are available from the corresponding author upon reasonable request.
